# Identification of Genomic Regions Influencing N-Metabolism and N-Excretion in Lactating Holstein- Friesians

**DOI:** 10.3389/fgene.2021.699550

**Published:** 2021-07-14

**Authors:** Hanne Honerlagen, Henry Reyer, Michael Oster, Siriluck Ponsuksili, Nares Trakooljul, Björn Kuhla, Norbert Reinsch, Klaus Wimmers

**Affiliations:** ^1^Genomics Unit, Institute for Genome Biology, Leibniz Institute for Farm Animal Biology (FBN), Dummerstorf, Germany; ^2^Metabolism Efficiency Unit, Institute of Nutritional Physiology “Oskar Kellner,” Leibniz Institute for Farm Animal Biology (FBN), Dummerstorf, Germany; ^3^Livestock Genetics and Breeding Unit, Institute of Genetics and Biometry, Leibniz Institute for Farm Animal Biology (FBN), Dummerstorf, Germany; ^4^Faculty of Agricultural and Environmental Sciences, University of Rostock, Rostock, Germany

**Keywords:** GWAS, emission, cattle, nitrogen, milk urea, non-urea nitrogen

## Abstract

Excreted nitrogen (N) of dairy cows contribute to environmental eutrophication. The main N-excretory metabolite of dairy cows is urea, which is synthesized as a result of N-metabolization in the liver and is excreted via milk and urine. Genetic variation in milk urea (MU) has been postulated but the complex physiology behind the trait as well as the tremendous diversity of processes regulating the N-metabolism impede the consistent determination of causal regions in the bovine genome. In order to map the genetic determinants affecting N-excretion, MU and eight other N-excretory metabolites in milk and urine were assessed in a genome-wide association study. Therefore phenotypes of 371 Holstein- Friesians were obtained in a trial on a dairy farm under near commercial conditions. Genotype data comprised SNP information of the Bovine 50K MD Genome chip (45,613 SNPs). Significantly associated genomic regions for MU concentration revealed *GJA1* (BTA 9), *RXFP1*, and *FRY1* (both BTA 12) as putative candidates. For milk urea yield (MUY) a promising QTL on BTA 17 including *SH3D19* emerged, whereas *RCAN2*, *CLIC5*, *ENPP4*, and *ENPP5* (BTA 23) are suggested to influence urinary urea concentration. Minor N-fractions in milk (MN) may be regulated by *ELF2* and *SLC7A11* (BTA 17), whilst *ITPR2* and *MYBPC1* (BTA 5), *STIM2* (BTA 6), *SGCD* (BTA 7), *SLC6A2* (BTA 18), *TMCC2* and *MFSD4A* (BTA 16) are suggested to have an impact on various non-urea-N (NUN) fractions excreted via urine. Our results highlight genomic regions and candidate genes for N-excretory metabolites and provide a deeper insight into the predisposed component to regulate the N-metabolism in dairy cows.

## Introduction

Dairy farming contributes with considerable amounts to total environmental nitrogen (N) emissions 2020 (Uwizeye et al., 2020). Due to societal demands and political decisions, the dairy sector is challenged to reduce its N-emissions in the near future. Generally, N-emissions in dairy farming arise after digestion and metabolization of dietary crude protein (CP) (Bryant, 1970; Abdoun et al., 2006). In detail, most of CP is digested by the microbiota to peptides, amino acids and ammonia (NH3). Depending on the availability of energy sources in the rumen, ammonia is utilized by microbes producing microbial protein (MOP) and facilitating microbial growth. The MOP and rumen undegradable protein are subsequently transported to and digested in the cow’s small intestine. The resulting amino acids and smaller peptides are absorbed by the intestinal epithelia. Non-digested dietary N, microbial N originating from the large intestine as well as endogenous N from the cow’s metabolism are excreted with feces (Nfec). Surplus ruminal ammonia is transported through the rumen epithelia and by blood proteins to the liver, where it is metabolized to urea by hepatocytes. Urea is excreted by urine and milk, or is transported via the blood stream across the rumen wall or saliva into the rumen. Ruminal urea is instantaneously converted to ammonia resulting in further MOP synthesis, independent of the dietary crude protein level (Munyaneza et al., 2017). Due to the dynamics of N-cycling, the concentrations of blood urea (BU), urinary urea (UU), and milk urea (MU) are directly correlated (Burgos et al., 2007). While quantitative measurements of UU excretions are difficult to carry out in practice, MU values are collected routinely in monthly milk records. Thereby MU is measured via infrared technology, which probably does not provide the highest accuracy but makes measurements available at a large scale. Thus, MU is considered a convenient parameter to assess N-excretion and N-use efficiency in large populations with multiple measurements per individual cow (Burgos et al., 2007, 2010).

In consequence of the cooperative N-usage between the cow and the microbes in the digestive tract, all N-excretions including MU, UU and Nfaec mean a loss of bioavailable N (Munyaneza et al., 2017). Besides urea, further N-fractions are excreted via urine and milk, comprising purine derivatives, creatine and creatinine, as well as hippuric acid and ammonia (Cox, 2013; Munyaneza et al., 2017). Although these non-urea-nitrogen (NUN) metabolites account only for a small part of total N-excretion, they also contribute to N-losses and N-emissions into the environment (Jardstedt et al., 2017). Generally, there is high variation in NUN-excretion (Cox, 2013), but also evidence for phenotypic variation in MU concentration, which is most commonly explained by the feed ration. Interestingly, Aguilar et al. (2012) referred to phenotypic variation in MU concentrations that could not be explained by ration composition and determined the cow itself as a highly significant factor influencing MU concentrations. The feed-independent variation in MU concentrations has led to the assumption that genetic components might have a significant impact on the N-metabolism and thus on N-excretion of dairy cows.

Indeed, several studies have calculated moderate heritabilities for MU concentrations in Holstein cows, ranging from 0.13 to 0.59 (Wood et al., 2003; Stoop et al., 2007; Stamer et al., 2011; Guarini et al., 2019). Average heritability estimates over a whole lactation comprised values from 0.24, 0.22 to 0.2 for Holsteins in first, second and third lactation, respectively (Jahnel et al., 2021). Estimations of the genetic relationship between MU concentrations and other milk production traits, such as milk-, fat-, and protein yield (Wood et al., 2003; Stamer et al., 2011; Höfener, 2019), and fat and protein percentage (Höfener, 2019) showed only little or even no correlation. Considering these facts, MU concentrations have a potential for breeding intervention based on genotypes that might determine a more efficient N-metabolism. Interestingly, a conventional breeding value for MU concentrations was calculated in consequence of political decisions in the Netherlands (Sebek et al., 2007) but not much has been published yet on the underlying genome regions. Only in a few studies, quantitative trait loci (QTL) for MU have been detected. For example, Bouwman et al. (2010) localized four genomic regions on BTA 1, 6, 21 and 23 for MU concentration and MU yield, using genotypes and test day data from 1926 Holstein-Friesians in the Netherlands. Cecchinato et al. (2014) detected three significantly associated SNPs on BTA 1, 7, and 26 in Brown Swiss cattle, while Pegolo et al. (2017) found regions on BTA 4, 5, and 13 significantly related to MU concentrations.

Apparently, the functional biodiversity and the quantitative character of MU make it difficult to determine genomic regions consistently associated with N-metabolism in dairy cows. Consequently, in the current study, in addition to the commonly investigated traits (MU concentration and MU yield), further N-excretion metabolites that are involved in N-metabolism were investigated in a genome-wide association approach. Specifically, the list of traits comprised MU, MU yield (MUY), urinary urea (UU), total minor N-metabolites in milk (MN) and four specific NUN-fractions in urine. The aim of our study was to uncover genetic variants and genomic regions that are significantly associated with MU and other N-excretion and N-metabolism traits in order to better understand the genetic background of N-metabolism in dairy cows.

## Materials and Methods

Animal husbandry and sampling were carried out according to the guidelines of the German Animal Protection Law. All protocols were approved by the Institute’s Animal Welfare Commission. The experimental protocol is in strict compliance with the German Animal Welfare Legislation, has been approved by the Ethics Committee of the federal state of Mecklenburg-Western Pomerania, Germany (Landesamt für Landwirtschaft, Lebensmittelsicherheit und Fischerei; LALLF M-V7221.3-2-019/19) and is in accordance with the ARRIVE guidelines.

### Cow Population

For this study, all 371 lactating Holstein-Friesians owned by the practice operating dairy farm Gut Dummerstorf (Mecklenburg-Western Pomerania, Germany) were investigated. The entire herd was housed in a free stall pen, fed twice daily (6.00 a.m./p.m., *ad libitum*) and milked twice daily (4.00 a.m./p.m.) in a 2 × 14 side-by-side milking parlor. All cows were fed the same ration, which provided 7.26 MJ NEL/kg dry matter and 15.4% crude protein resulting on average in a slightly negative ruminal nitrogen balance of –0.9 g N/kg dry matter. The composition of the ration was constant throughout the trial. The cows were in 1st to 9th lactation and at least 21 days in milk. The average herd’s daily milk yield was 36.2 kg per day with an average lactation duration of 364 days. The cows descended from 145 sires and 163 dam sires.

### Sample Collection and Analysis

The trial was carried out in four subsequent sampling time points in spring 2020. Milk samples were obtained as pooled samples of morning (25 ml) and evening milking (25 ml) for each cow in the monthly milk record procedure (in total 50 ml per cow). Individual urine samples (10 ml) were collected in a maximum time distance of 24 h relative to the monthly milk record procedure (*n* = 371). The cows’ body weights were recorded two times during the entire trial right after the morning milking.

Milk constituents were measured by the State Control Federation of Mecklenburg-Western Pomerania. From each cow, the pooled milk sample (50 ml) was conserved in a Bronopol/Kathon mixture and stored at 4°C for not longer than 48 h before urea content was determined using mid infrared spectroscopy (CombiFoss 7, Foss, Hilleroed, Denmark). Therefore, milk samples were homogenized and heated to 40°C and the absorption measured at the corresponding wavelength was electronically transformed on the basis of a regression calculation (Partial Least Squares) into MU concentrations (mg/l). Furthermore, data including protein, fat, lactose and milk yield of monthly milk records were available from the herd.

In addition, the total N content of milk was analyzed by MQD (Qualitätsprüfungs- und Dienstleistungsgesellschaft Mecklenburg-Vorpommern, Güstrow, Germany) according to the Kjeldahl method in accordance with ASU §64 LFGB L 01.00–10/1. Results were converted to milk crude protein (g/100 g), using the factor 6.38.

Urine samples were obtained right after morning or evening milking by dorso-ventral stimulating massages, ventral of the vulva and during spontaneous urinating. The urine was immediately stored in 15 ml tubes on ice, before it was frozen at –20°C. Frozen urine samples were thawed on ice, homogenized and centrifuged at 13,000 × g and 4°C for 5 min before they were analyzed for urea and NUN-metabolites. Urea was diluted 20-fold in water and analyzed photometrically at a ABX Pentra C400 clinical chemistry analyzer (HORIBA Europe GmbH, Oberursel, Germany). The NUN-metabolites were measured by HPLC (1200/1260 infinity Series; Agilent Technologies, Waldbronn, Germany) as previously described (Müller et al., 2021). Detection and quantification of NUN-metabolites were conducted at 230 nm with exception for allantoin and creatine at 210 nm.

### Phenotypic Traits

Nine phenotypes, divided into repeatedly measured traits (RMTs) and single measured traits (SMTs), were generated from milk and urine data. RMTs represent the entire cow individual lactation by using the available milk record data of 14 months. SMTs are derived from one-time measurements during the sampling trial. RMTs using milk data are MU in mg/l and MUY in g. For MU, values of 14 months milk record data were averaged for each cow. MUY was generated by multiplying the individual daily milk yield with the MU value of each test day in the 14 months period, summed up and averaged. Milk nitrogen (MN) is the only single-measured milk trait and contains all N-fractions in the milk, i.e., protein-N and non-protein-N (NPN) in g/100 g milk.

All urine traits represent SMTs, expressed in mmol/l urine. Urinary traits comprise UU and the purine derivatives allantoin (AL) and its precursor uric acid (UA). Furthermore, hippuric acid (HA) and creatinine (CRE) as well as its precursor creatine (CR) were quantified.

### Genotype Data

The whole cow population of Gut Dummerstorf was genotyped in the course of the German-wide genotyping project KuhVision (Rensing et al., 2017). SNP chip data of the project was imputed by vit Verden (Vereinigte Informationssysteme Tierhaltung, Verden, Germany) on the Bovine 50K MD Genome chip (45,613 SNPs). The imputing was conducted as part of the genetic evaluation routine with 825,999 Holstein cows and pedigree information of 2,467,138 animals, using FIMPUTE version 3.0 (Sargolzaei et al., 2010). The imputing accuracy per chromosome was reported to be 99.5–99.7% by vit Verden. Probe sequences of the SNP chip were mapped to the current bovine genome assembly and the annotation of markers was updated to ARS-UCD1.2 (access 01 September 2020). Markers not mapping to autosomes in ARS-UCD1.2 were discarded (1,940 SNPs). Furthermore, genotype data was filtered for minor allele frequency (MAF) > 0.05 and deviation from Hardy Weinberg equilibrium (*p* > 1 × 10^–6^) (4,220 SNPs discarded). After imputing, mapping and filtering, 39,453 SNPs remained for genome-wide association analysis.

### Genome-Wide Association Study (GWAS)

A two-step approach was utilized to identify genomic regions associated with nitrogen phenotypes in milk and urine conducting the GAPIT R package with the BLINK method and default parameters (Lipka et al., 2012; Huang et al., 2019). In a first step, all phenotypes were adjusted for environmental effects in a linear model, giving residuals for each trait. The phenotypes were adjusted as follows: For MU and MUY, the average milk fat content of the lactation was included as covariate in the model accounting for differences in feed intake between individuals, considering the slightly negative RNB in the feed ration. For MU, lactation number, which separates cows in their first lactation from other cows, was considered as fixed effect in the model to correct for physiological protein anabolism in the course of growth during first lactation. Lactation number was not included in the model for MUY, due to a confounding with milk yield, which is a component of MUY calculation. For MN, milk protein and MU content of the test day were integrated as covariates in the model to investigate only the NPN-and NUN-fractions in milk. In addition, for MN and for UU, the milk fat content of the test day was included as covariate to account for variation in feed intake. For all urinary traits, except for CRE, the covariates CRE and body weight were used to correct for differences in urine volume. CRE was adjusted for deviation in muscle metabolism with body weight as covariate.

In a second step, GWAS was performed with the trait residuals by using the Bayesian information and Linkage-disequilibrium Iteratively Nested Keyway (BLINK) method. BLINK executes two fixed effect models in an iterative approach. One of which accounts for population stratification by testing each SNP one by one with multiple associated SNPs that are fitted as covariates on the testing SNP. The second model selects the covariate SNPs instead of a kinship matrix to correct on the relationship in the population investigated. With this approach, BLINK abolishes the necessity for the genes underlying a trait to be evenly distributed along the genome in order to enhance statistical power (Huang et al., 2019). For each trait, a quantile-quantile (QQ) plot was generated, based on the observed *P*-values.

To account for linkage disequilibrium between markers, the R script SimpleM was used to estimate the number of independent tests. SimpleM calculates a composite linkage disequilibrium matrix from the SNP genotypes and uses this matrix in a principal component analysis to determine the effective number of independent tests (Gao et al., 2008). For the data set 20,517 independent tests were ascertained. The significance thresholds were consequently set at 1/20,517 [–log10 (*P*-value) = 4.31] for suggestive significance and at 0.05/20,517 [–log10 (*P*-value) = 5.61] for genome-wide significance (Lander and Kruglyak, 1995). Genome-wide results for each trait were visualized in Manhattan plots.

### Data Integration and Candidate Genes

Based on the results from GWAS, the genomic regions surrounding the significant SNPs were examined for each trait. In accordance with Saatchi et al. (2014), 1 Mb regions were defined around the significant SNPs (500 kb each direction from the SNP) as putative QTL regions. The QTLs were manually investigated for the two genes closest to the right and left of the significant SNPs and for positional (covering significantly associated SNP) and functional (postulated function with relevance to the trait) candidate genes. Genomic information and functional annotations of genes were extracted from GeneCards^1^ and Ensembl^2^.

## Results

Both the milk and urine traits showed appreciable variation in the Holstein population studied (Table 1). However, urine traits showed a considerably higher variability compared to milk traits. This might be due to varying urine volumes, which led to a dilution or concentration of the urine components (Spek et al., 2012). QQ plots from GWAS are shown in Supplementary Figure 1 for all traits, indicating that the approach used was suitable to control for population stratification.

**TABLE 1:** Descriptive statistics of milk and urinary nitrogen traits in Holstein cows.

Acronym	Trait	Unit	Matrix	Analysis	Min	Max	Mean	SD	N
MU	Milk urea	mg/l	Milk	RMT^a^	121.00	274.54	192.53	24.80	371
MUY	Milk urea yield	g	Milk	RMT	3.64	10.53	6.84	1.37	371
UU	Urinary urea	mmol/l	Urine	SMT	13.40	308.00	102.54	49.63	331^c^
MN	Milk nitrogen	g/100 g	Milk	SMT^b^	2.70	4.75	3.62	0.38	368^d^
UA	Urine acid	mmol/l	Urine	SMT	0.40	3.06	1.52	0.31	331^c^
AL	Allantoin	mmol/l	Urine	SMT	1.59	31.68	18.01	5.48	331^c^
HA	Hippuric acid	mmol/l	Urine	SMT	5.99	60.70	27.04	9.93	331^c^
CR	Creatine	mmol/l	Urine	SMT	0.49	12.61	3.64	1.80	331^c^
CRE	Creatinine	mmol/l	Urine	SMT	0.75	9.35	4.35	1.59	331^c^

*^*a*^RMT, repeated measurement trait.*

*^*b*^SMT, single measurement trait.*

*^*c*^Reliable body weight measurements could only be recorded in 331 out of 371 cows, resulting in 331 phenotypes for urine traits since body weight was included as covariate in the urine models.*

*^*d*^Three samples out of initially 371 had been discarded in laboratory analysis resulting in 368 MN phenotypes*

### Urea in Milk and Urine

**FIGURE 1:**
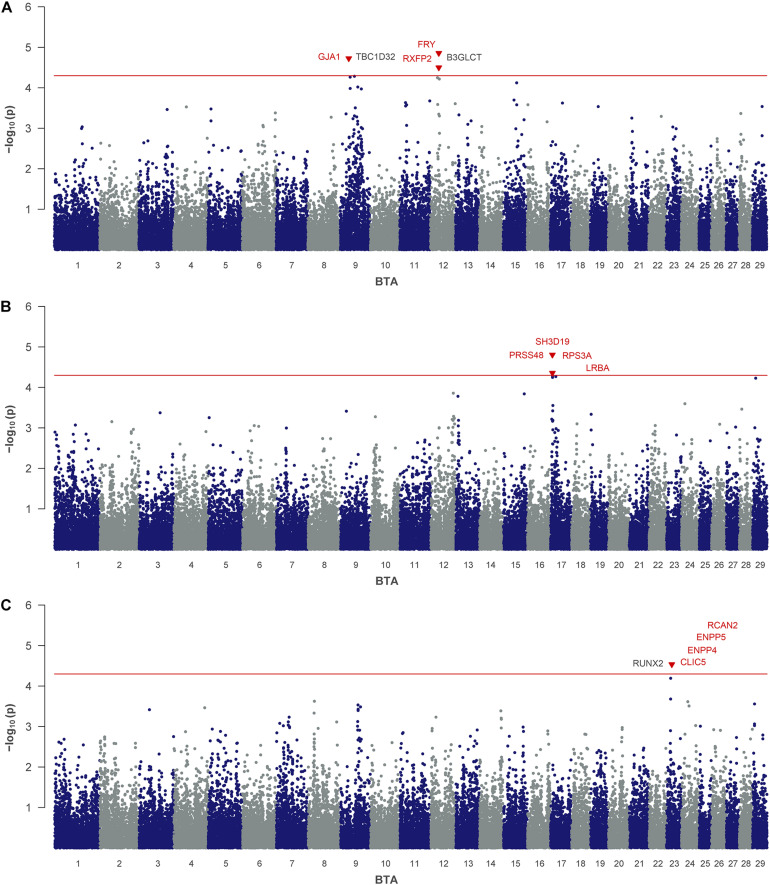
Genome-wide association analysis of urea traits: **(A)** milk urea, **(B)** milk urea yield and **(C)** urinary urea. The red line indicates the threshold for suggestive significance. Genes with a suggested positional or biological association to the trait are indicated in red. Genes, located closest to the right and left of the lead SNP in a 1-Mb window are colored in black.

For MU, three significant SNPs were identified, which mapped on BTA 9 (rs41609177) and on BTA 12 (rs110792428, rs110799535) (Table 2 and Figure 1A). On BTA 12 both significant SNPs are located in the same QTL region between 28.8 and 29.9 Mb, which comprises six putative candidate genes. The region on BTA 9 indicated at 29.8 Mb contains the annotated genes gap junction protein alpha 1 (*GJA1*) and domain family member 32 (*TBC1D32*).

**TABLE 2:** Overview of QTLs and suggested candidate genes for N-traits identified by GWAS.

Trait	BTA^a^	QTL interval (Mb)^b^	Significant SNP	–log_10_ (*P-*value)	MAF^c^	*R*^2^d^	Candidate gene^e^
MU	9	29.29–30.29	rs41609177	4.73	0.26	0.06	*GJA1, TBC1D32*
MU	12	28.84–29.89	rs110792428	4.50	0.42	0.06	*RXFP2, FRY*
			rs110799535	4.86	0.41	0.06	
MUY	17	6.12–7.15	rs41835093	4.81	0.31	0.07	*SH3D19, PRSS48, LRBA, RPS3A*
			rs41835125	4.36	0.38	0.06	
UU	23	18.49–19.49	rs109244808	4.54	0.14	0.05	*CLIC5, RCAN2, ENPP4, ENPP5*
MN	4	20.65–21.65	rs43375405	4.91	0.44	0.05	
MN	16	57.55–58.85	rs41814676	4.52	0.10	0.04	*ASTN1*
			rs41814682	4.52	0.10	0.04	*PAPPA2*
MN	17	18.57–19.57	rs41576712	5.89	0.188	0.06	*SLC7A11*
MN	17	17.56–18.56	rs109749663	4.85	0.06	0.05	*MGST2, ELF2*
UA	7	63.30–64.30	rs42372803	5.45	0.36	0.06	*NMUR2*
HA	7	38.87–39.87	rs109848970	8.82	0.07	0.09	
HA	12	74.36–75.36	rs43706910	5.96	0.32	0.05	
HA	17	69.78–70.78	rs41634411	5.59	0.37	0.06	*SFI1*
CR	4	116.16–117.16	rs109873598	4.37	0.24	0.05	*DPP6*
CR	5	82.0–83.00	rs110770413	4.85	0.31	0.06	*ARNTL2, ITPR2*
CR	5	65.16–66.16	rs41590238	4.83	0.06	0.06	*MYBPC1*
CR	6	46.21–47.21	rs43705592	4.31	0.32	0.06	*STIM2*
CR	7	63.44–64.44	rs110536156	5.12	0.40	0.06	
CR	7	67.16–68.16	rs42332347	4.53	0.39	0.05	*SGCD*
CR	18	23.62–24.62	rs109756131	4.74	0.41	0.06	*SLC6A2*
CRE	16	2.51–3.51	rs41787830	4.35	0.15	0.06	*TMCC2, MFSD4A*

*^*a*^Bos taurus autosome.*

*^*b*^Compiled according to Saatchi et al. (2014).*

*^*c*^Minor allele frequency of the significant SNP.*

*^*d*^Calculated by linear regression between phenotype residuals and SNP-alleles.*

*^*e*^Selected due to a positional or functional association to the trait; MU, milk urea concentration; MUY, milk urea yield; UU, urinary urea; MN, minor N-fractions (non-protein, non-urea) in milk; UA, uric acid; HA, hippuric acid; C, creatine; CRE, creatinine.*

For MUY as the consolidating trait of milk urea and milk yield, rs41835093 and rs41835125 were found to be significantly associated (Table 2 and Figure 1B). The two SNPs mapped on BTA 17 at 6.6 Mb, only 0.03 Mb apart from each other. They are located in the positional candidate gene SH3 domain containing 19 (*SH3D19*). Furthermore, in close vicinity six other genes are annotated in this QTL region.

For UU, as the quantitatively most relevant nitrogen fraction in urine, rs109244808 on BTA 23 indicated a single QTL at 18.9 Mb (Table 2 and Figure 1C). The QTL harbors five genes, including chloride intracellular channel 5 (*CLIC5*) and regulator of calcineurin 2 (*RCAN2*).

### Non-urea-N (NUN) in Milk and Urine

**FIGURE 2:**
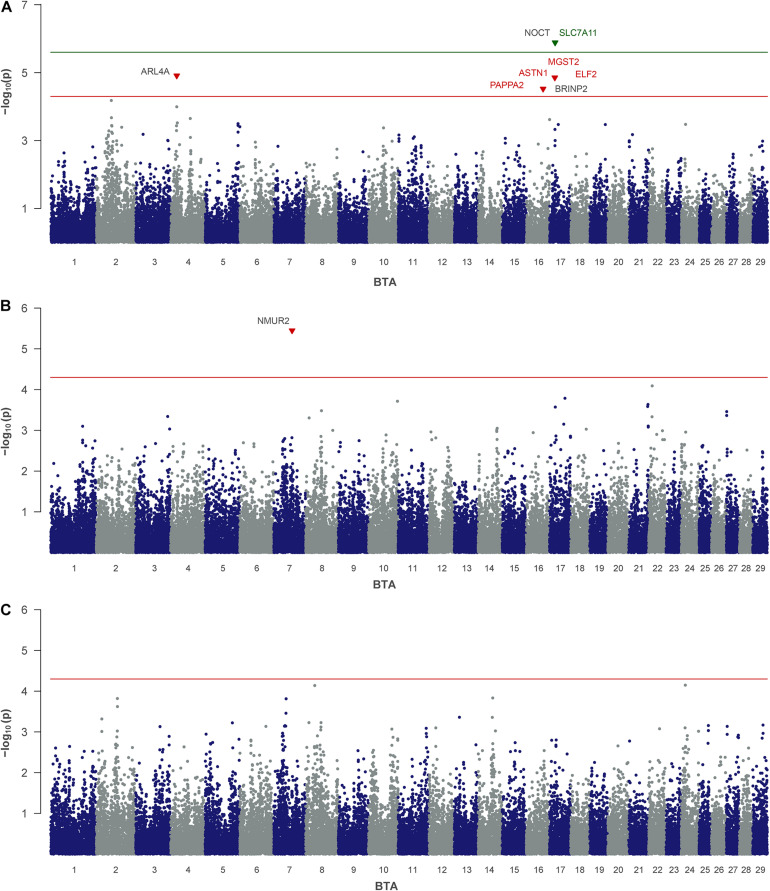
Genome-wide association analysis of non-urea-nitrogen traits: **(A)** milk nitrogen, **(B)** uric acid and **(C)** allantoin. The red and green lines indicate suggestive and genome-wide significance thresholds, respectively. Genes with a suggested positional or biological association to the trait are indicated in red and green. Genes located closest to the right and left of the lead SNP in a 1-Mb window are colored in black.

For MN, which represents minor NPN and NUN-metabolites in milk, QTLs on BTA 4, 16, and 17 were indicated by five significantly associated SNPs (Table 2 and Figure 2A). One of them, rs41576712 on BTA 17, exceeded genome-wide significance. The same chromosome harbors a further QTL region, with rs109749663 at 18.06 Mb highlighted as significantly associated. This SNP indicates microsomal glutathione S-transferase 2 (*MGST2*) as positional candidate. On BTA 16, rs41814676 and rs41814682 mapped only 0.03 Mb apart from each other in astrotactin 1 (*ASTN1*) and pappalysin 2 (*PAPPA2*) at 58 Mb. The significantly associated SNP on BTA 4 at 21.15 Mb points on ADP ribosylation factor like GTPase 4A (*ARL4A*) and scinderin (*SCIN*) as putative candidate genes in the vicinity.

For AL no SNP exceeded the significance threshold, but for UA one SNP occurred significant (Table 2 and Figures 2B,C). This SNP (rs42372803) is located on BTA 7 at position 63.8 Mb and represents an intron variant of the long non-coding RNA ENSBTAG000054682. However, in close proximity, neuromedin U receptor 2 (*NMUR2*) provides a putative functional candidate gene.

**FIGURE 3:**
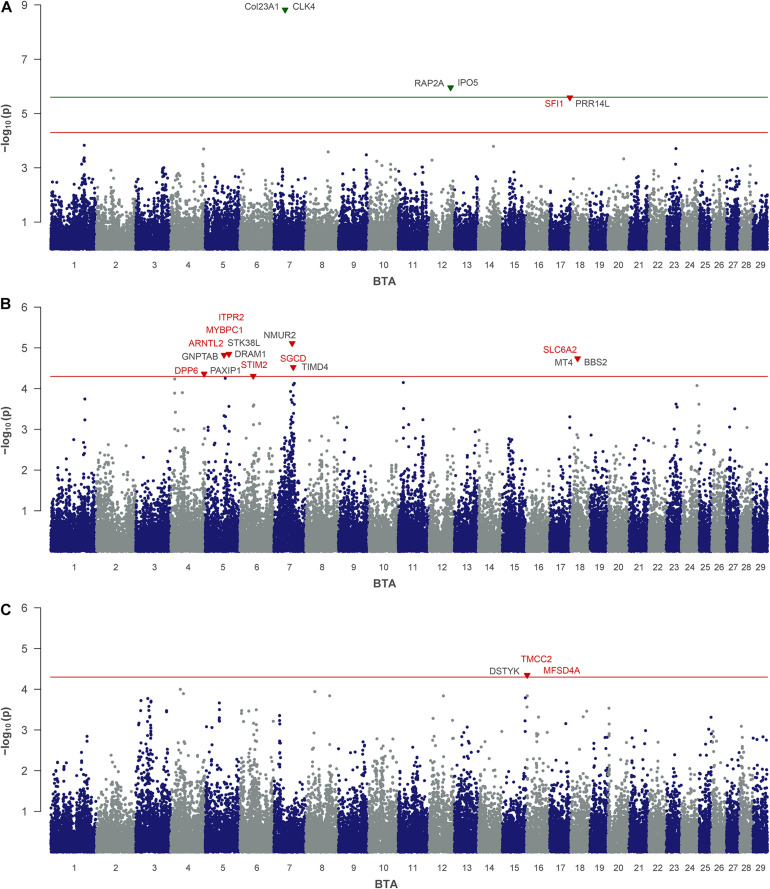
Genome-wide association analysis of non-urea-nitrogen traits: **(A)** hippuric acid, **(B)** creatine and **(C)** creatinine. The red and green lines indicate suggestive and genome-wide significance thresholds, respectively. Genes with a suggested positional or biological association to the trait are indicated in red. Genes, located closest to the right and left of the lead SNP in a 1-Mb window are colored in black.

For HA, as a by-product of phenolic acid digestion excreted by urine, the analysis revealed two SNPs that reached the genome-wide significance threshold. These are rs109848970 (*P* = 8.82) on BTA 7 and rs43706910 (*P* = 5.96) on BTA 12 (Table 2 and Figure 3A). Although neither of these two SNPs directly mapped in a gene region, rs109848970 opens up a QTL at 39.4 Mb (BTA 7) with 15 annotated genes. In the immediate vicinity of rs43706910 at 74.86 Mb on BTA 12 six genes are annotated so far. A third SNP on BTA 17 (rs41634411) was significantly associated with HA. This SNP is located at 70.28 Mb in the gene encoding for the SFI1 centrin binding protein (*SFI1*).

For CR, which plays an indispensable role in muscular energy metabolism, the urinary levels in the cow herd were significantly associated with seven SNPs located on BTA 4, 5, 6, 7, and 18 (Table 2 and Figure 3B). Two QTLs on BTA 5 and one QTL on BTA 18 revealed more than 10 annotated genes, respectively, while the other QTL regions included less than five annotated genes, each. Three significantly associated SNPs, that are rs110770413 (BTA 5 at 82.5 Mb), rs42332347 (BTA 7 at 67.7 Mb) and rs109873598 (BTA 4 at 116.7 Mb) mapped in gene regions and indicated aryl hydrocarbon receptor nuclear translocator like 2 (*ARNTL2*, BTA 5), sarcoglycan delta (*SGCD*, BTA 7) and dipeptidyl peptidase like 6 (*DPP6*, BTA 4) as positional candidates.

For CRE, which is an excretory derivative of CR, one SNP (rs41787830) on BTA 16 reached the threshold of suggestive significance (Table 2 and Figure 3C). This SNP is located at 3.0 Mb in an intron of the gene coding for transmembrane and coiled-coil domain family 2 (*TMCC2*). Furthermore, this QTL includes another 16 annotated genes.

## Discussion

As a part of the effort to reduce N-emissions in dairy cows, this work analyzed the genetics of several relevant traits that contribute to the variance in N-excretion. For this purpose, a herd of 371 Holstein cows was comprehensively phenotyped for nine N-fractions from milk and urine and investigated in a GWAS approach. Significantly associated genomic regions and potential candidate genes were identified for eight of these fractions.

### Urea in Milk and Urine

N-excretion is generally influenced by the cow and its rumen microbiome. The most relevant N- excretion fraction in terms of quantity is urea (Munyaneza et al., 2017). Besides the microbiological impacts in the rumen, mainly the following processes are conceivable to influence the amount of excreted and recycled nitrogen in the cow: (i) absorption of ammonia via the rumen epithelia, transport to the liver by blood proteins and hepatic synthesis of urea; (ii) transport of synthetized urea from liver via blood (BU) to kidney, udder and rumen; (iii) diffusion and excretion of BU via nephrons (UU) and the udder epithelium (MU).

For MU, significant genomic regions were identified on BTA 9 and 12. The region on BTA 9 indicates *GJA1* as a potential candidate. *GJA1* encodes connexin 43, a component of gap junctions that contribute to the diffusion of low molecular weight substances into cells (Graham and Simmons, 2005). Therefore, it seems conceivable that *GJA1* also influences the diffusion of urea across the udder epithelium, and thus the urea content of the milk. Interestingly, connexin 43 was previously shown to play a critical role in the development of the mammary gland epithelia and in lactation processes in mice and cattle (Plante and Laird, 2008; Farhadian et al., 2021). Moreover, *GJA1* has been associated with fertility parameters in cows (Ribeiro et al., 2016; Neupane et al., 2017). In the second QTL identified for MU on BTA 12, a number of candidate genes related to cellular turnover processes are mapped and annotated. Interestingly, the udder epithelia of cows is subject to strong cellular turnover events during lactation. Specifically, in early lactation massive cell proliferation causes the increase in milk yield, while apoptosis in late lactation strongly influences the dry period induction (Stefanon et al., 2002; Hale et al., 2003). Considering that cellular turnover processes have an impact on the diffusion capacities in the udder epithelium, an influence of those genes on MU levels seems to be conceivable. In this context relaxin family peptide receptor 2 (*RXFP2*) was shown to be significantly associated with mammary gland morphology in Simmental cattle (Pausch et al., 2016). Moreover, FRY microtubule binding protein (*FRY*) has been confirmed to influence the development and function of the mammary gland (Liu et al., 2019).

Milk urea yield was calculated as the absolute MU volume over the entire lactation period, i.e., even with a lower MU per liter, a cow with a high milk yield can have a comparatively high MUY. Two significant SNPs on BTA 17 revealed *SH3D19* as positional candidate for MUY. Although *SH3D19* has not been postulated in the context of urea or milk yield so far, it has been mentioned in several studies concerning fertility traits in cattle and pigs (Kranc et al., 2017; Guarini et al., 2019; Brazert et al., 2020). Fertility traits and milk yield basically compete for available energy in early lactation of high-yielding cows. Genomic correlations between milk yield and fertility parameters as well as milk urea and fertility traits have already been proven by several studies (König et al., 2008; Mucha and Strandberg, 2011; Sawa et al., 2011). It is also worth noting that other genes in this QTL such as serine protease 48 (*PRSS48*), LPS responsive beige-like anchor protein (*LRBA*) and ribosomal protein S3A (*RPS3A*) have also been mentioned in the context of fertility. In fact, *RPS3A* serves as an indispensable factor in reproductive processes not only in cattle but also in sheep (He et al., 2017; Pokharel et al., 2020). It is therefore conceivable that not only the positional candidate *SH3D19* but the entire QTL has an influence on fertility, milk urea and milk yield (Hu et al., 2019). More detailed investigations of this genomic region toward the influence on energy metabolism as the common denominator of MUY and fertility traits are proposed.

For UU a single QTL on BTA 23 revealed six candidate genes. Four of which (*RCAN2*, *CLIC5*, *ENPP4*, and *ENPP5*) are of relevance due to their biological functions in the nephron. Filtration and secretion processes in the nephron determine the amount of excreted UU. Both, the filtration of urea in the glomerus, the first section of the nephron, and the reabsorption of urea back into the blood in the last section of the tubule, depend on concentration-dependent diffusion. *RCAN2*, which regulates the release of calcineurin, may be an obvious candidate for UU because calcineurin activates podocyte (glomerus cell) apoptosis (Wang et al., 2011). This might affect the absolute renal diffusion rate for urea in the glomerus, resulting in an altered amount of urea in urine. *CLIC5* is indispensable for the development and preservation of glomerular endothelial cells and podocytes. In addition, chloride transporters in general play an important role in establishing the concentration gradient between blood and urine, which is relevant for filtration and reabsorption of urea (Schwiebert et al., 1994; Jentsch et al., 2002). Finally, ectonucleotide pyrophosphatase phosphodiesterase 4 and 5 (*ENPP4* and *ENPP5*) might play a key role in the activation of purinoceptors, which are known as renal tubular transporters regulating sodium and water balance (Vekaria et al., 2006). Sodium influences the amount of reabsorbed urea by its osmotic potential, while the water balance is influenced by the amount of reabsorbed urea.

### Non-urea-N (NUN) in Milk and Urine

Apart from urea, several other N-metabolites are excreted via milk and urine. These NUN are mainly intermediates and end products of protein and nucleic acid degradation and are known to be highly variable between individuals (Bristow et al., 1992).

Milk nitrogen generally comprises all N-fractions in milk including proteins, urea and NUN. By calculating the residuals considering the effects of MU and milk protein in the model, the present GWAS for MN focuses on the NUN-fractions in milk. A major subset of NUN in milk are free amino acids (Roginski et al., 2003; Rafiq et al., 2016). Among the four QTLs identified for MN, three significantly associated SNPs mapped in *ASTN1*, *PAPPA2*, and *MGST2*. According to current annotations, these genes are involved in neuroblast development, energy metabolism and glutathione metabolism. While *MGST2* has been associated to milk fat metabolism in dairy cows (Gebreyesus et al., 2019), a link to variations in NUN-fractions in milk has not been postulated for any of these three genes so far. However, in the same QTL region, E74 like ETS transcription factor 2 (*ELF2*) seems to be more likely to explain individual variation in MN. *ELF2* has been determined as a major transcription factor driving the formation of milk protein in high yielding dairy cows (Maas et al., 1997). Specifically, *ELF2* mediates the translation of mRNA to protein and thus limiting the rate of milk protein synthesis. Milk protein is generally synthesized by the lactocytes utilizing the influx of amino acids. Unused amino acids in this process are excreted as part of MN. Thus, increased or decreased milk protein synthesis induced by *ELF2* could have a major impact on MN. The bioavailability of limiting amino acids is a further determinant of milk protein synthesis. Solute carrier family 7 member 11 (*SLC7A11*) on BTA 17 encodes for a highly specific amino acid transport system that enables the influx of extracellular cysteine in exchange for intracellular glutamate (Verrey et al., 2004). An impact of *SLC7A11* on amino acid availability might influence milk protein synthesis and thus determining the amount of unused amino acids excreted via MN. In the QTL on BTA 4, neither *ARL4A* nor *SCIN* have been associated with minor N-fractions so far, but *ARL4A* has been postulated as a candidate gene for milk yield, recently (Vijayakumar et al., 2019).

Uric acid (UA) is a subsequent product of purine nucleotide degradation. Precisely, the purine nucleotides are degraded to xanthine and oxidized via xanthine oxidoreductase to UA mainly in the intestinal mucosa and in the liver of cattle (Gonzalez-Ronquillo et al., 2003). Although in ruminants, the purine influx mainly derives from intestinal digestion and absorption of rumen microbial nucleic acids (Balcells et al., 1991) UA is excreted constantly, independent of the microbial protein synthesis and the feeding ration (Susmel et al., 1995). One QTL on BTA 7 has been significantly associated with UA, revealing *NMUR2* in the vicinity. NMU, the encoded neuropeptide receptor, has been attributed a central role in the regulation of feeding behavior, feed intake and body weight in animal models (Howard et al., 2000; Sampson et al., 2018), but an association to UA synthesis or excretion in cattle has not been postulated so far.

No genomic region has been significantly associated with allantoin (AL) excretion in urine. AL is a subsequent product of oxidized UA and the quantitatively highest excretion metabolite of purine bases in cows’ urine (Bristow et al., 1992; Cox, 2013). It is known that variance in AL is primarily influenced by the rate of microbial protein synthesis (Kehraus et al., 2006; Tas and Susenbeth, 2007).

Hippuric acid (HA) results from the hepatic conjugation of glycine and benzoic acid. The latter is a product of phenolic acid fermentation in the rumen (Sun et al., 2020). Interestingly, HA content in manure could have a significant impact on N-losses from the soil, making it a valuable trait for further genetic improvement, in addition to nutritional measures to reduce N-emissions (Gardiner et al., 2016). The QTL on BTA 7 contains several genes with functional annotations in extracellular matrix formation and degradation (*Col23A1*, *B4GALT7*, *PHYKPL*), in nuclear ribosomal processes (*HNRNPAB*, *NHP2*, *RMND5B*) and in intracellular transports to the Golgi complex (*TMED9*). The significantly associated SNP in the QTL on BTA 12 mapped between RAP2A, member of RAS oncogene family (*RAP2A*) and importin 5 (*IPO5*). The former gene links intestinal microvilli formation and cell polarization, whereas *IPO5* enables the transport of proteins into the nucleus (Gloerich et al., 2012). Based on the current annotation, the proposed genes provide no direct link to influence the variability in HA. However, the rumen microbiota might have an impact on HA differences by synthesizing divergent benzoic acid levels, but if the significantly associated genes influence the ruminal microbial composition and thus affect benzoic acid and HA levels asks for further research.

Creatine (CR) is synthesized in the liver and acts as an energy storage for nucleoside phosphates in the muscle. Since the muscular energy metabolism is regulated by a dynamic variety of biological processes, a polygenic influence on CR levels seems conceivable. *SGCD* on BTA 7 and myosin binding protein C1 (*MYBPC1*) on BTA 5 are involved in muscle anabolism and muscle contraction. The protein encoded by *MYBPC1* influences the skeletal muscle contractility by targeting muscle-type creatine kinase to myosin filaments. Furthermore, inositol 1,4,5-trisphosphate receptor type 2 (*ITPR2*) on BTA 5 and stromal interaction molecule 2 (*STIM2*) on BTA 6 play roles in Ca^2+^ muscle storage and release. Creatine phosphate levels are known to influence Ca^2+^ entries in skeletal muscle fibers (Fryer et al., 1995). *ITPR2* encodes a receptor that mediates the mobilization of intracellular Ca^2+^ stores, while STIM proteins act as Ca^2+^ sensors and influence the Ca^2+^ influx. In addition, the solute carrier family 6 member 2 (*SLC6A2*) gene localized in the QTL on BTA 18 is of interest due to the identification of a causal mutation for creatine transporter deficiency in humans (Schiaffino et al., 2005).

Creatinine (CRE) is the hydrolyzed derivative of CR and the main excretion product of creatine in urine. A single SNP on BTA 16 had been significantly associated with CRE, indicating *TMCC2* as positional candidate. *TMCC2* forms complexes with the apolipoprotein E and thus influences proteolytic pathways (Hopkins et al., 2011). Given the fact that CR influences muscle turnover processes, a link to protein degradation is conceivable. Furthermore, major facilitator superfamily domain containing 4A (*MFSD4A*) is involved in the opening of glucose dependent sodium channels in the medulla of the kidney and thus influencing the osmotic gradient between tubule and interstitium. Urea reabsorption in the kidney is dependent on both this osmotic gradient and the opening of sodium channels. Interestingly, higher urinary CRE concentrations have been observed in cows with lower urine and milk urea concentrations (Müller et al., 2021). An interplay of urea and CRE excretion via *MFSD4A*, in the context of total N-excretion might therefore be further explored.

## Conclusion

This study proposes a number of genomic regions and candidate genes associated with MU concentration and a further eight N-excretory traits in milk and urine of Holstein-Friesians. The results complement the knowledge of genetic determinants regulating the N-metabolism of dairy cows, which influences in its entirety N-emissions in dairy farming. In the light of a sustainable food production the results can be of relevance for a targeted exploitation of predisposed physiological plasticity in N-metabolism of dairy cows.

## Data Availability Statement

The data was provided by the German Evaluation Center (VIT, Verden) and is subject to the following licenses/restrictions: the data is proprietary and cannot be released publicly. Requests to access these datasets should be directed to the authors.

## Ethics Statement

The animal study was reviewed and approved by the Ethics Committee of the Federal State of Mecklenburg-Western Pomerania, Germany (Landesamt für Landwirtschaft, Lebensmittelsicherheit und Fischerei; LALLF M-V7221.3–2–019/19.

## Author Contributions

HH organized the sampling trial. HH and HR carried out the sampling trial, performed statistical analysis and interpretation of the data, and wrote the manuscript. NT, MO, and SP supported data analysis and interpretation. NR, BK, and KW jointly designed and supervised the study, and contributed to the interpretation of the data, and to the writing of the manuscript. All authors reviewed the final manuscript.

## Conflict of Interest

The authors declare that the research was conducted in the absence of any commercial or financial relationships that could be construed as a potential conflict of interest.

## Footnotes

1 http://www.genecards.org

2 http://www.ensembl.org
