# Supplementary figures and images for: Identification of Genomic Regions Influencing N-Metabolism and N-Excretion in Lactating Holstein- Friesians
Source: Front Genet. 2021 Jul 14;12:699550. doi: 10.3389/fgene.2021.699550 (PMC8318802; doi:10.3389/fgene.2021.699550)

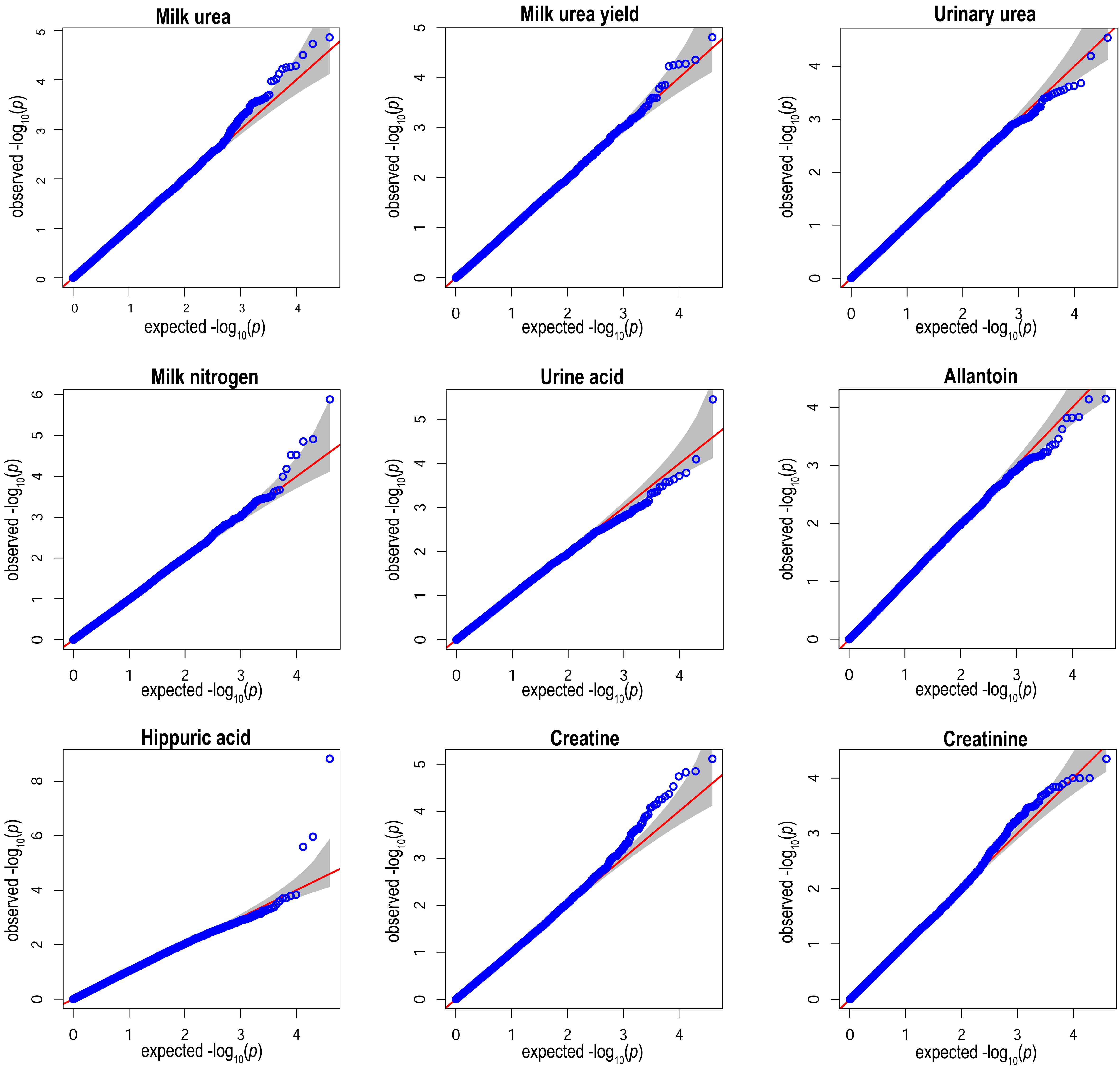

Supplement: Supplementary file 1 [file Image_1.TIF]
